# A new interpretation of nonpulmonary vein substrates of the left atrium in patients with atrial fibrillation

**DOI:** 10.1002/joa3.12521

**Published:** 2021-02-22

**Authors:** Mariano Rillo, Zefferino Palamà, Raffaele Punzi, Salvatore Vitanza, Angelo Aloisio, Silvia Polini, Antonella Tucci, Annalisa Pollastrelli, Francesco Zonno, Antonio Anastasia, Cesare Franco Giannattasio, Luigi My

**Affiliations:** ^1^ Arrhythmology Service Division of Cardiology ‐ Casa di Cura Villa Verde Taranto Italy; ^2^ Abbott Medica Italia srl Sesto San Giovanni (Mi) Italy

**Keywords:** 3D mapping, ablation index, atrial fibrillation, catheter ablation, lesion index

## Abstract

**Background:**

Substrate analysis of the left atrium in patients undergoing atrial fibrillation ablation has limitations when performed by means of simple bipolar acquisition.

**Objective:**

To evaluate the incidence of low‐voltage zones (LVZs) through maps constructed by means of various catheters: multipolar (MC), omnipolar (OC), and circular catheters (CMCs) with the 3D electro‐anatomical systems (3d‐S) CARTO3 and EnSite Precision.

**Methods:**

To assess LVZs, we acquired maps by means of CMC and MC in the voltage range 0.05‐0.5 mV in 70 consecutive patients in sinus rhythm. In the case of OC only, we made an intra‐patient comparison of bipolar maps constructed by means of the along and across, and HD‐Wave configurations of the EnSite 3d‐S in the ranges of 0.05‐0.5 and 0.5‐1.0 mV. On the basis of this comparison, we chose the range that best identified LVZs as a set of different colors (SDC) compatible with patchy fibrosis (qualitative analysis). Subsequently, we detected the voltage values corresponding to purple and gray points, close to SDC, and the value inside corresponding to blue, green, and red colors, and we evaluated the color change in other voltage ranges. Finally, we performed a quantitative analysis of LVZs by applying the qualitative characteristics described above.

**Results:**

On the basis of our settings, for OC, the optimal range identifying LVZs was 0.3‐0.6 mV. OC revealed smaller LVZs than MC (*P* < .05 or *P* < .001), except in the lateral wall. No significant differences were observed between CMCs.

**Conclusions:**

In our experience, OC does not present the limits of bipolar HD maps, though further studies are needed in order to confirm that 0.3‐0.6 mV is the optimal voltage range within which to identify LVZs.

## Graphical abstract

This project aims to evaluate the incidence of low voltages through maps constructed by means of various catheters: multipolar (MC), omnipolar (OC), and circular catheters (CMCs) with the 3D electro‐anatomical systems (3d‐S) using an optimized voltage range (0.3‐0.6 mV) to identify better LVZs.

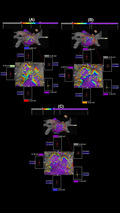

## 1 INTRODUCTION

Although pulmonary vein (PV) isolation (I) remains a cornerstone of any atrial fibrillation (AF) ablation,^1^, ^2^, ^3^ other anatomical regions of the left atrium, that is, non‐PV substrates, are involved in AF, especially in persistent AF.^4^, ^5^ High‐density (HD) endocardial voltage mapping by means of multipolar (MC) catheters and 3d electro‐anatomical systems (3d‐S) has been increasingly used in clinical practice to identify both left ventricular^6^ or left atrial anatomical areas of low‐voltage electrical activity,^7^, ^8^ which is commonly considered a marker of atrial fibrosis.^9^ Left atrial substrate modification by targeting low‐voltage zones (LVZs) is an ablation strategy that, in addition to pulmonary vein isolation (PVI), tries to erase arrhythmogenic mechanisms harbored in such tissue.^7^, ^10^ However, this approach has limitations because LVZs are subject to various influences, such as the bipole orientation expressed by the angle of attack and the activation wave‐front; this can mean that electrical signals may not be recorded even when they are present^11^. Recent reports have described experiences of the use of new catheters with omnipolar (OC) recording capacity, which do not seem to be affected by the negative influences described above with regard to bipolar HD maps.^12^, ^13^ The aim of the present study was to perform a post hoc analysis in patients undergoing PVI, in order to evaluate the incidence of non‐PV substrates detected by different diagnostic catheter technologies, including MC, OC, and circular mapping catheters (CMCs), and by means of qualitative and quantitative analyses of LVZs on applying various voltage ranges.

## 2 METHODS

### 2.1 Study population

Between February 2019 and January 2020, we enrolled 70 consecutive patients undergoing PVI for paroxysmal AF (n = 40, 57.14%) or persistent AF (n = 30, 42.87%). All patients were strongly symptomatic for palpitations, fatigue, dyspnea, or chest pain, and refractory to antiarrhythmic drugs (AADs). Table 1 reports their baseline characteristics at the time of ablation. All patients provided written informed consent after being adequately informed of the risks and benefits. After PVI, we performed HD mapping in all patients during sinus rhythm: in 34 patients, we used the CARTO® mapping System (CARTO^®^ 3 V6, Biosense Webster, Inc) and the Lasso^®^ Nav Circular Catheter or the PentaRay^®^ Nav Multipolar Catheter (Biosense‐Webster, Inc); in 36 patients, we used the EnSite Precision™ 3d system (Abbott) and the Inquiry™ AFocus II™ Circular Catheter or Advisor™ HD Grid‐Mapping Catheter (Abbott).

**TABLE 1:** Baseline characteristics of patients at the time of ablation

Row labels	All patients	PAF	PsAF	PAF vs PsAF *P* value
	(n = 70)	(n = 40)	(n = 30)	
Age (SD)	64.5 ± 8.9	64,5 ± 9	64 ± 9	*P* =.20
Male	47 (67.1%)	25 (62.5%)	22 (73.3%)	
Hypertension	58 (82.8%)	30 (75%)	28 (93.3%)	*P* =.04
Diabetes	14 (20%)	5 (12.5%)	9 (30%)	*P* =.07
Obese (BMI ≥ 30)	18 (25.7%)	7 (17.5%)	11(36.7%)	*P* =.47
Overweight (BMI ≥ 25)	45 (64.3%)	28 (70%)	17 (56.7%)	*P* =.19
CAD	10 (14.3%)	6 (60%)	4 (40%)	*P* >.5
Type of AF		40 (57.2%)	30 (42.8%)	
AF duration				
≥48 m	27 (38.6%)	14 (35%)	13 (43.3%)	*P* =.07
≤36 m	40 (57.1%)	23 (57.5%)	17 (43.5%)	*P* =.72
LVEF ≥ 30%	8 (11.43%)	4 (50%)	4 (50%)	*P* =.07
LVEF ≥ 50%	62 (88.57%)	36 (58%)	26 (42%)	*P* =.01
LA volume (ml/m^2^)				
≥28	37 (53%)	13 (35%)	24 (65%)	*P* <.0001
<28	33 (47%)	27 (82%)	6 (18%)	*P* <.0001

Note

Values are expressed as numbers and percentages.

Abbreviationss: AF, Atrial Fibrillation; BMI, body mass index; BSA, body surface area; CAD, coronary artery disease; LA, Left Atrium, LVEF, Left Ventricular Ejection Fraction; m, months; ml/m^y^, milliliters per square meter; PAF, paroxysmal AF; PsAF, persistent AF; SD, standard deviation.

### 2.2 Procedural setup: Ablation settings

PVI was carried out through contact force‐guided ablation by means of a 3.5‐mm open irrigated‐tip catheter SmartTouch (Biosense Webster's ThermoCool^®^ SmartTouch^®^, Biosense Webster, Inc) or TactiCath (Abbott). Surface electrocardiographic leads (aVF, V_1_ and V_6_) and bipolar intracardiac electrograms filtered at 30 to 500 Hz were recorded on a recording system Prucka CardioLab EP v6.9.0 Recording System (GE Healthcare).

### 2.3 Voltage mapping

**FIGURE 1:**
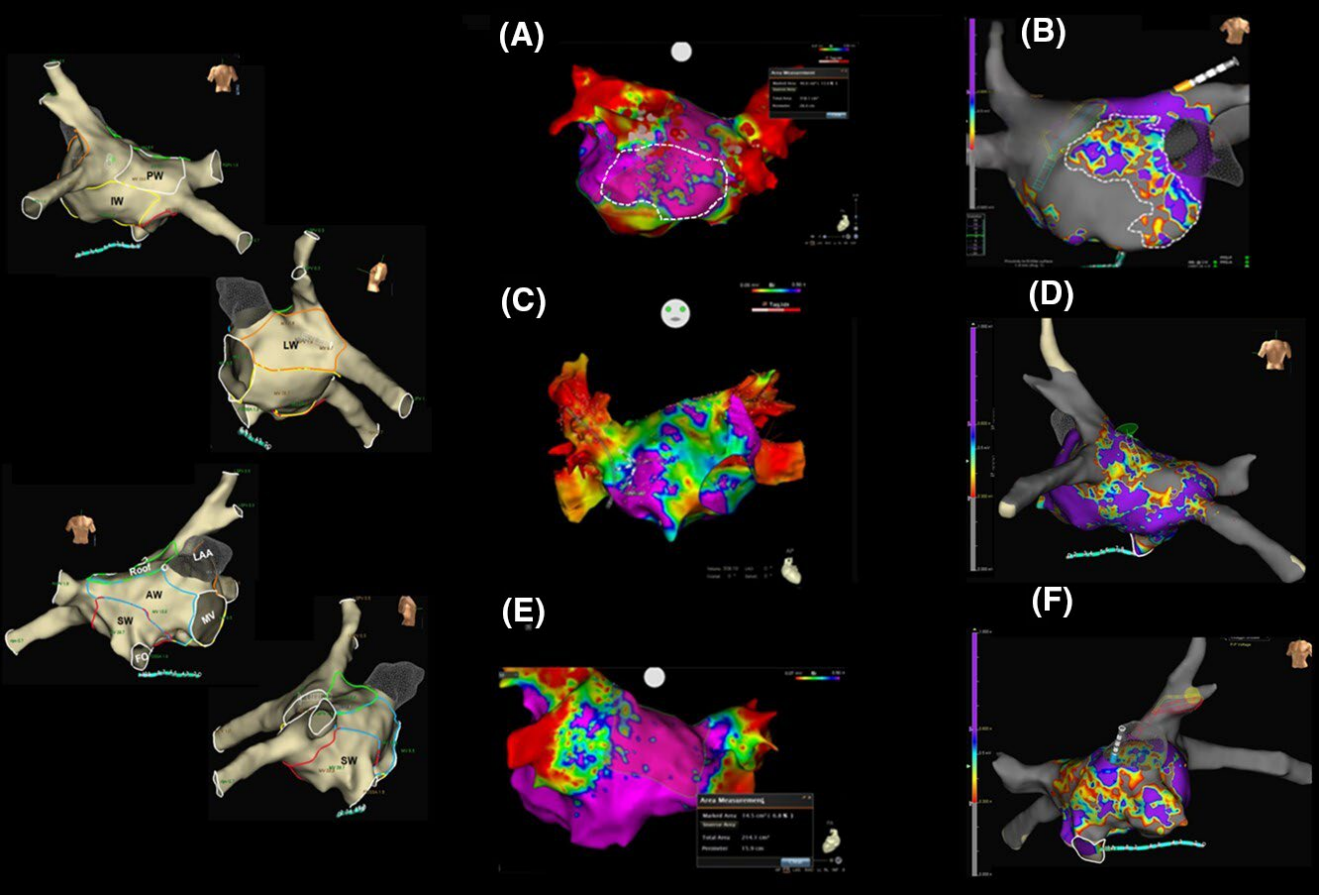
Qualitative and quantitative analyses of low‐voltage zones (LVZs): HD maps acquired by means of OC and MC. *Left panel*: the anatomical areas acquired with the 3d electro‐anatomical systems (3d‐S). *Right panel*: A, C, and E show the maps acquired by means of the multipolar catheter and 3d‐S CARTO 3, while B, D, and **F** show the maps acquired by means of the omnipolar catheter and 3d‐S EnSite Precision. A, PA projection. Persistent AF and dilated left atrium (LA). Map with 2.520 points acquired in the 0.05‐0.5 mV voltage range. The inferior wall (IW) presents LVZs, characterized by small islands of blue/green/red colors (potential patchy fibrosis) within a larger area of healthy tissue (purple). According to our results, this map could be influenced by the limitations of bipolar recordings. B, AP Projection. Persistent AF and dilated LA. Map with 28.000 points acquired in the 0.3‐0.6 mV voltage range. The entire anterior wall (AW) presents LVZs. A large area of different colors (dashed area) is clearly visible; this is characterized by small islands of normal tissue (purple), separated by probable patchy fibrosis (blue/green/yellow/red) inside the remaining AW of gray color; this latter area displays no electrical activity and is unable to mediate reentry. C, AP projection. Persistent AF and nondilated LA. The entire AW presents LVZs and is green in color (compatible with dense fibrosis) with only some small islands of healthy tissue (purple) and scar tissue (red), which poorly represent patchy fibrosis. From our point of view, this substrate is unlikely to be able to mediate reentry. However, this map could also be influenced by the limitations of bipolar recordings. D, PA Projection. Paroxysmal AF and nondilated LA. The posterior wall (PW) presents LVZs that is qualitatively compatible with patchy fibrosis. E, PA Projection. Persistent AF and dilated LA. The IW presents normal tissue, and the PW a few small islands of low voltage which are not able to mediate reentry. F, AP Projection. The same patient as in D. The map shows a prevalence of green and red colors that are more compatible with dense fibrosis and less so with patchy fibrosis, indicating a substrate that is probably unable to mediate reentry

**FIGURE 2:**
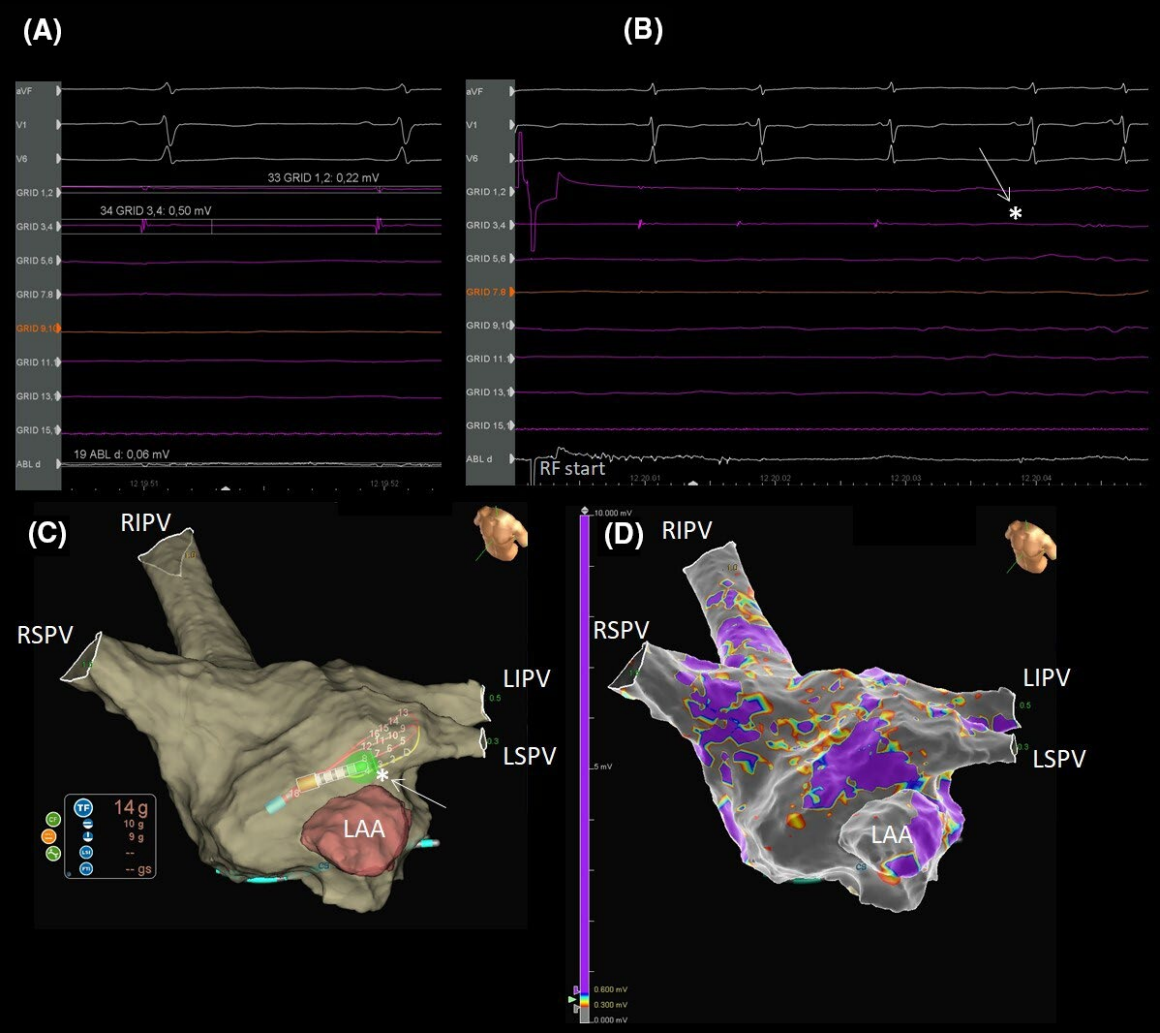
Direct comparison of bipolar versus omnipolar recordings. A, Pair 3, 4 of the HD Grid records a 0.5 mV signal, while the signal recorded by pair 1, 2 is of 0.22 mV. The distal dipole of the ablation catheter records a signal of 0.06 mV. B, During PVI complete disappearance of the HD Grid signal at the fourth beat after the beginning of RF (white arrow and star). C, The 3d anatomical map. Note the contact force of the ablation catheter equal to 14 grams during PVI (white arrow and star). D, The related electro‐anatomical map.

After PVI, the patients underwent HD mapping of the lesions around PVs and of the entire left atrium. All HD maps were constructed during sinus rhythm, either spontaneous or restored by electrical cardioversion. The left atrial anatomy was subdivided into six non‐PV regions: anterior wall, lateral wall, inferior wall, septal wall, posterior wall, and left atrial roof. The boundaries of each area were defined as shown in Figure 1. Any LVZs of the left atrium were classified according to their location. In 34 patients, HD maps were acquired by means of the CMC Lasso Nav (17) or the MC Pentaray (17) and the Coloring/Confidence mapping algorithm of the 3d‐S CARTO, which enables automated map acquisition. We performed a qualitative analysis of each HD map acquired, based on the standard voltage range reported in the literature (0.05‐0.5 mV), to assess LVZs localized in non‐PV anatomical areas of the left atrium. For this purpose, we searched for numerous small islands of low voltage characterized by a set of red/yellow/orange/green/light‐blue/blue colors (SDC), which we considered diseased left atrial tissue (patchy fibrosis), scattered within larger areas of electrical signals >0.5 mV of purple color (healthy tissue) or for small islands of healthy tissue within larger LVZs ≤ 0.5 mV of blue/green/red color, considered dense fibrosis, or of no electrical activity (only red color:scar tissue) ≤0.05 mV. Lesions compatible with dense fibrosis or scar tissue around PVs were one of the criteria on which PVI was based. All HD maps were based on the acquisition of as many voltage points as possible, and the Tissue Proximity Indicator filter of 3d‐S CARTO was used to exclude map points deemed not to be in contact with the shell. Figure 1A,C,E shows HD maps acquired by means of 3d‐S CARTO. In 36 patients, HD maps were acquired by means of the CMC AFocus (18) or a 16‐electrode HD configuration with 3 mm spacing by means of the standard Advisor OC HD Grid (18). For both types of catheter, the best duplicate algorithm of 3d‐S EnSite Precision was used; this creates voltage maps using the electrogram with the highest amplitude in the same position when multiple points are projected, while the worst voltage signals are automatically discarded, each time the catheter passes a point. We evaluated tissue contact using the Proximity Indicator of the 3d‐S EnSite Precision and exploiting the flexibility of the OC, which folds when it comes into contact with the wall. The voltage range used to characterize the substrate with the CMC AFocus was 0.05‐0.5 mV. Qualitative analysis was performed in the same way as for the maps acquired with 3d‐S CARTO. Only in the case of the OC did we try to identify an optimal voltage range for the definition of non‐PV substrates. To do so, in each patient with LVZs in the maps constructed by means of the OC (11/18 patients), we compared the bipolar maps in the “along and across” configuration of the 3d‐S EnSite Precision with the OC maps in the HD‐wave configuration; in this operation, we used two contiguous voltage ranges: 0.05‐0.5 and 0.5‐1 mV. Indeed, the 3D‐S EnSite Precision allows us to change the voltage range even after completing and saving the maps and the patient has left the electrophysiology laboratory, and subsequently to compare the new maps (post processing evaluation). We used a total of 33 HD‐wave and bipolar maps for the intra‐patient comparison in each range considered. Anatomical areas of gray color were considered to be scar tissue and those of purple color to be normal tissue. On the basis of the results of the comparison of the two voltage ranges, we chose the range more able to identify LVZs characterized by SDC. Furthermore, in order to demonstrate that LVZs identified in this way represents true non‐PV substrate, we measured on this latter map the voltage of the electrical signals corresponding to a purple point and a gray point among those closest to the SDC area. We also measured the voltage of a red point (lower voltage value), a green point (intermediate voltage value), and a blue point (higher voltage value) within the SDC area. All values are the result of the sum of the peak value of positive and negative voltage. In addition, we further evaluated the changes observed in the SDC area in other intermediate voltage ranges between 0.5 and 1.0 mV and still able to identify SCD, evaluating the relative color of the same points analyzed in the starting range. All HD maps were based on the acquisition of as many voltage points as possible. Finally, in all patients with non‐PV LVZs identified by means of both 3d‐S CARTO and 3d‐S EnSite Precision, and with qualitative characteristics of HD maps compatible with potential patchy fibrosis identified as described above, we also tried to quantify the extent of LVZs as a percentage of the surface area of the six different left atrial anatomical regions; these were calculated in square centimeters from the measurements yielded by the two 3D mapping systems. Figure 1B,D,F shows the HD maps acquired by means of 3d‐S EnSite Precision. OC signals were occasionally compared with the bipolar signals acquired through the distal dipole of the ablation catheter positioned in contact with the electrodes of the HD Grid, detecting electrical activity. However, these comparisons were not used for evaluations in this study. Figure 2 shows an example of direct comparison.

### 2.4 Postablation management and follow‐up

The patients were followed up for a period of about 12 months. The protocol included the prescription of AAD: for a 3‐month blanking period in patients with paroxysmal AF and without interruption in patients with persistent AF. Patients underwent clinical follow‐up examinations 3, 6, 9, and 12 months after the procedure. AAD was resumed in patients with PAF in the event of recurrence. A 24‐hour Holter recording was performed at the scheduled controls. Anticoagulant therapy was continued indefinitely on the basis of the CHADS‐VASc score (≥2 in men and ≥3 in women).

### 2.5 Statistical analysis

Continuous variables are expressed as mean ± SD, and categorical variables as percentages. The Student's *t* test and Pearson's Chi‐square test were used to compare continuous and dichotomous variables, respectively. We constructed Kaplan‐Meier curves to illustrate 1‐year freedom from AF recurrence, using a log‐rank test. Two‐tailed tests were considered statistically significant at the level of 0.05. All analyses were performed by means of STATA 13.1 (STATA Corp.).

## 3 RESULTS

### 3.1 Patients

The two groups of patients (with paroxysmal and persistent AF) were homogeneous in terms of age, sex, diabetes, coronary artery disease, and body mass index (Table 1). Hypertension and left atrial dilation were more frequent in persistent than in paroxysmal AF patients (*P* =.04 and *P* <.001, respectively), while left ventricular ejection fraction >50% and normal left atrium were more frequent in paroxysmal than in persistent AF (*P* = .01 and *P* <.001, respectively; Table 1). Kaplan‐Meier curves demonstrated that the freedom from AF was comparable in all patients (AFocus vs Lasso Nav *P* =.6; OC vs MC *P* =.8).

### 3.2 Procedural data

**FIGURE 3:**
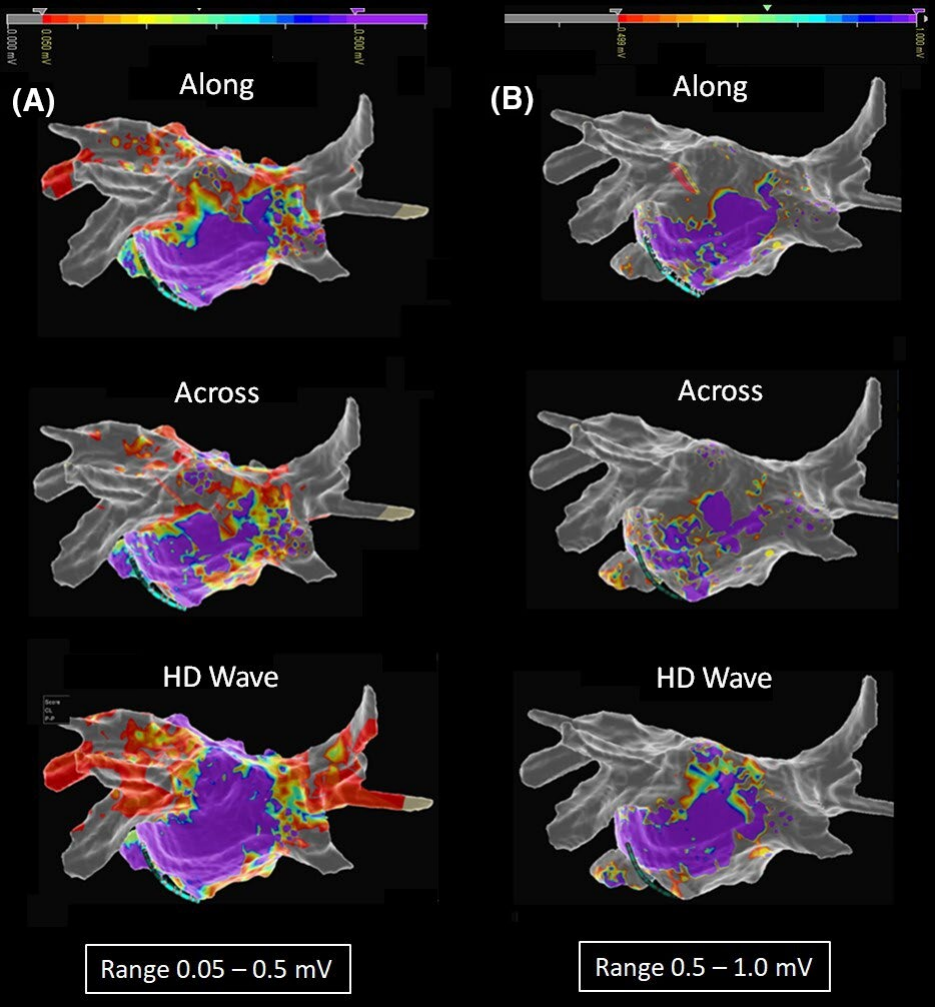
Intra‐patient HD map: comparison of along, across, and HD‐wave maps in the two basic range. A, PA projection. Qualitative analysis after setting 0.05‐0.5 mV voltage range. Bipolar maps in along and across configuration showed LVZs in the posterior wall, which we considered compatible with patchy fibrosis (set of different colors), while in the inferior wall only normal tissue (purple color) was observed in the along configuration and partially patchy fibrosis in the across configuration. Finally, omnipolar maps in the HD‐wave configuration showed normal tissue in both the posterior and inferior walls. Note the gray color in the bipolar map, which demonstrates PVI, and the red color in the omnipolar map, which seems not to completely prove PVI. B, Re‐evaluation after setting 0.5‐1.0 mV voltage range. The along and across bipolar HD maps showed LVZs increase, with a shift to gray (transformation from potential patchy fibrosis into scar). The HD‐wave map also showed LVZs increase, but with a shift from purple to a set of different colors (transformation from normal tissue to potential patchy fibrosis). Note PVI in both bipolar and HD‐wave maps (gray color in both)

**FIGURE 4:**
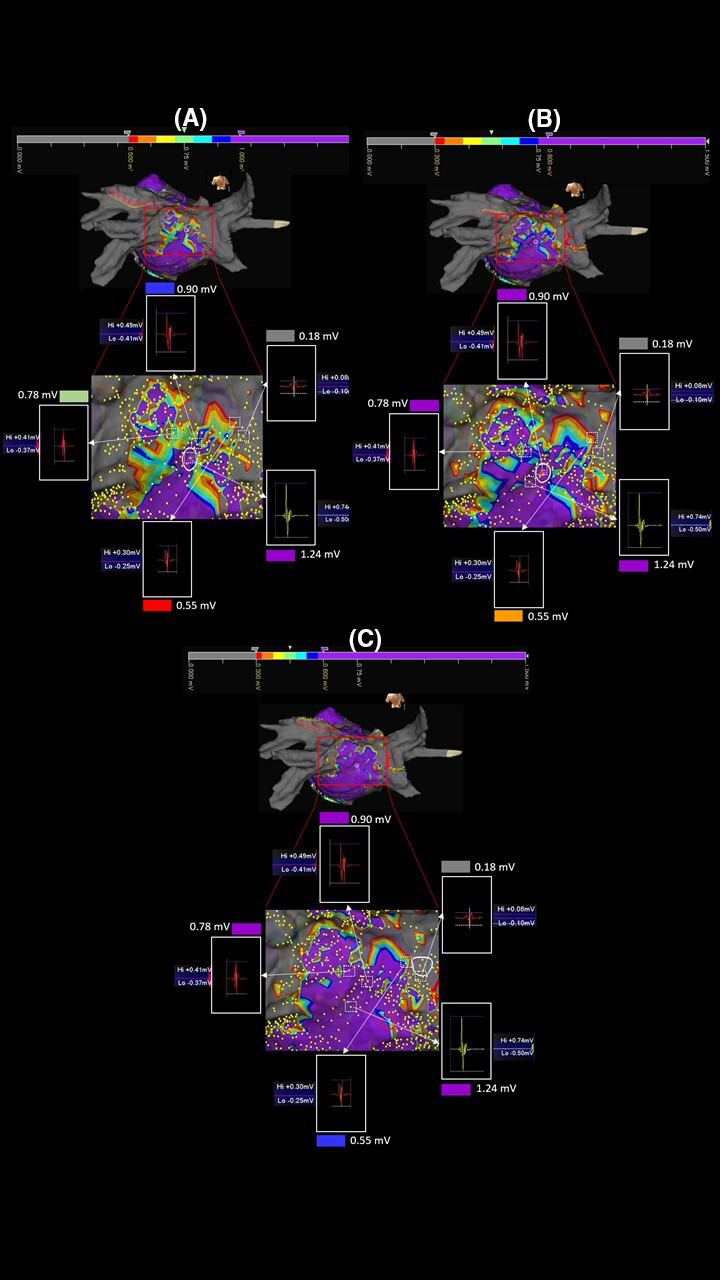
Editing maps by changing the basic range. Maps of the same patient reported in Figure 3. Comparing the three ranges A: 0.5‐1.0, B: 0.3‐0.8, and C: 0.3‐0.6 mV, a progressive reduction of LVZs characterized by a set of red/orange/yellow/green/light‐blue/blue colors (SDC) can be noted with minimal representation in the range 0.3‐0.6 mV. The white squares with discontinuous lines and the relative white arrows identify the points under analysis. The voltage of the points is unchanged in the three maps demonstrating that the analyzed points are the same. The color change instead demonstrates a misleading identification of LVZs expression of potential patchy fibrosis in the ranges different from 0.3 to 0.6 mV

**FIGURE 5:**
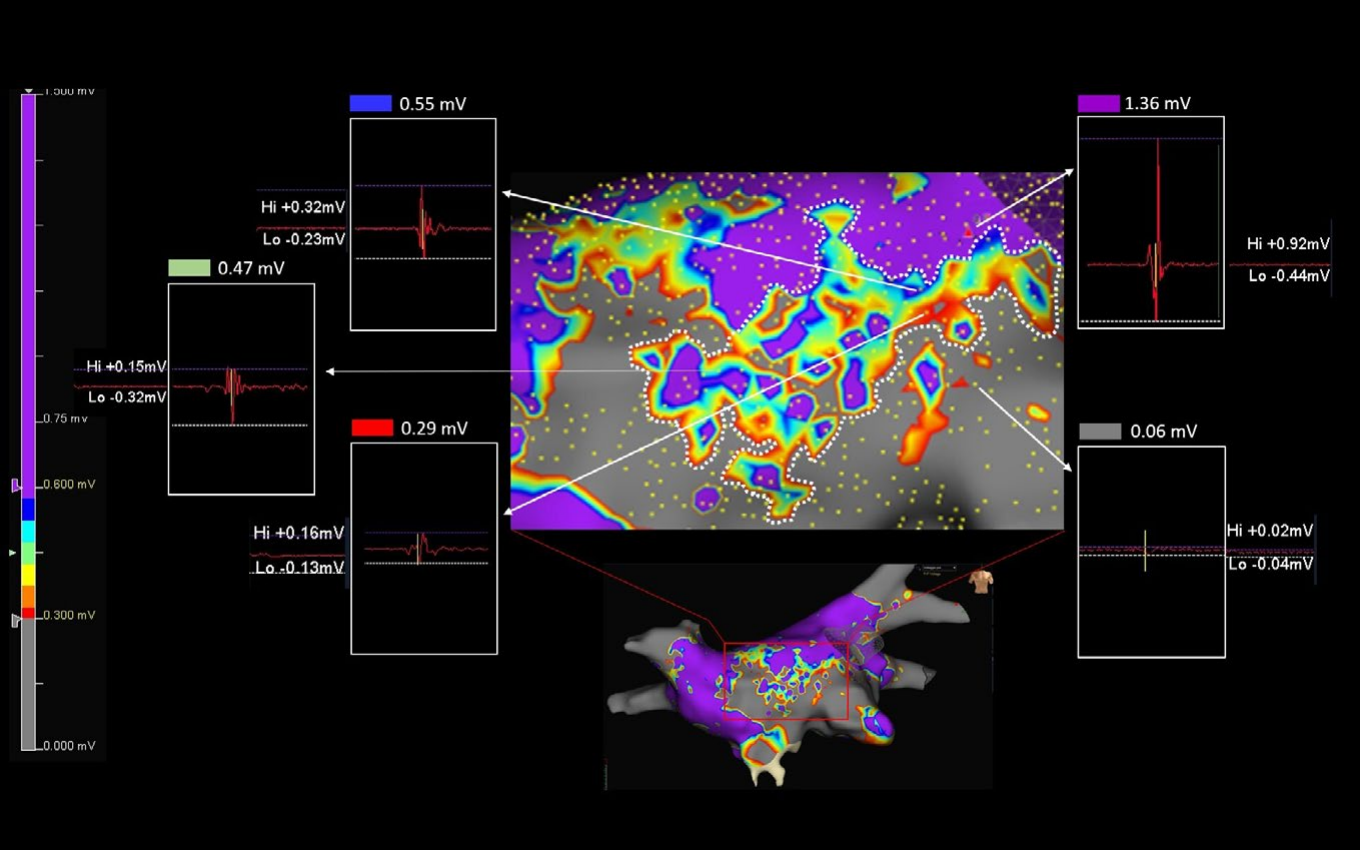
Choice of the optimal range within which to identify potential patchy fibrosis. Magnification of a map acquired with the 0.3‐06 mV range in another patient of our series. In this case, the minimum voltage value of a gray point and maximum of a purple point in close proximity to the SDC were 0.06 and 1.36 mV, respectively, while inside the SDC minimum value was 0.29 mV (red point), intermediate value was 0.47 mV (green point), and maximum value was 0.55 mV (blue point). Note that the voltage value of each point is the sum of positive peak value (Hi) plus the negative one (Lo). Each analyzed point is identified by the white arrows. The values measured in the SDC are within the new range of 0.3‐0.6 mV we are using to identify potential patchy fibrosis

Complete PVI with bidirectional conduction block was achieved in all 70 patients by means of pacing maneuvers and HD mapping. The procedural time, including HD mapping, was 126 ± 13 minutes in patients who underwent PVI and mapping by CMC Lasso Nav or MC (*P* >.5), and 119 ± 15 minutes in patients in whom CMC AFocus or OC (*P* >.5) was used for mapping. The numbers of voltage points acquired were: 1583 ± 431 with CMC AFocus versus 1501 ± 317 with Lasso Nav (*P* >.5), and 18 626 ± 4617 with OC versus 2037 ± 343 with MC (*P* <.0001). In our intra‐patient comparison of along and across bipolar maps, we observed both an increase in LVZs on passing from the 0.05‐0.5 mV range to the 0.5‐1 mV range and a shift from SDC to gray (transformation of areas characterized by potential patchy fibrosis into scar) in all 11 patients re‐evaluated (100%). Comparison of the 22 OC maps (HD‐wave maps) also showed an increased LVZs in 100% of patients re‐evaluated when the 0.5‐1.0 mV range was used, but with a shift from purple to SDC (transformation of areas characterized by normal tissue into potential patchy fibrosis). An example of these comparisons is shown in Figure 3, demonstrating that for OC map only in the range 0.5‐1 mV SDC were identified. Figure 4 shows the analysis of the points near and inside the SDC in a patient of our series. The analysis of points close to the SDC in the starting range of 0.5‐1.0 mV highlighted signals of 1.24 mV for the purple color and 0.18 mV for the gray color. The color of these points remained unchanged in maps with ranges 0.3‐0.8 mV and 0.3‐0.6 mV. The analysis of the points inside the SDC in the starting range of 0.5‐1.0 mV highlighted values of 0.90 mV for the blue color, 0.78 mV for the green color, and 0.55 mV for the red color. The color of the three points under analysis, however, changed in the range 0.3‐0.8 and 0.3‐0.6, since we observed a progressive reduction in the extension of the SDC area and an increase in the purple area. In particular, in the range 0.3‐0.8 mV, the blue and green points have become purple and the red color has become orange; in the range 0.3‐0.6 mV, the blue and green points of the 0.3‐0.8 mV range remained purple and the orange point became blue. Since in the range 0.3‐0.6 mV we observed the minimum extension of the SDC, other ranges were excluded from the evaluation. On the basis of these results, we defined the optimal voltage range, which was between 0.3 and 0.6 mV, and used this value in our further evaluations (Figure 5). The average of the values expressed in mV in these patients studied and emerged from the analysis of the points near the SDC performed in the orthogonal maps with a range of 0.3‐0.6 mV was 1.5 ± 0.35 mV for the purple color and 0.09 ± 0.05 mV for the gray color. For the points analyzed within the SDC in the maps using the same range 0.3‐0.6 mV, the average value was 0.50 ± 0.03 for the blue points, 0.41 ± 0.05 for the green points, and 0.33 ± 0.02 for the red points.

### 3.3 Comparison of patients with and without non‐PV LVZs

Table 2 shows our comparison of patients with and without non‐PV LVZs. LVZs were found in 38 of 70 patients (54.3%). From the comparison between HD maps, no statistically significant differences emerged in terms of the number of patients with LVZs between CMC AFocus and CMC Lasso Nav (*P* =.725), or between OC and MC (*P* =.332). No LVZs were found in 32 of 70 patients (45.7%), nor did any statistically significant differences emerge in terms of patients without LVZs between CMC AFocus and CMC Lasso Nav (*P* =.725), or between OC and MC (*P* =.332).

**TABLE 2:** Patients with and without LVZs

Patients	Tot. N.	AFocus	Lasso Nav	*P*	HD Grid	Pentaray	*P*
All patients	70	18 (25.7%)	17 (24.3%)		18 (25.7%)	17 (24.3%)	
LVZs	38 (54.3%)	5 (13.3%)	8 (21%)	.73	11 (28.9%)	14 (36.8%)	.33
none LVZs	32 (45.7%)	13 (40.6%)	9 (28.1%)	.24	7 (21.9%)	3 (9.4%)	.16

Note

Values are expressed as numbers and percentages.

Abbreviations: LVZs, low‐voltage zones; SD, standard deviation; VP, voltage points.

### 3.4 Comparison of LVZs: CMC AFocus versus Lasso Nav and OC versus MC

**FIGURE 6:**
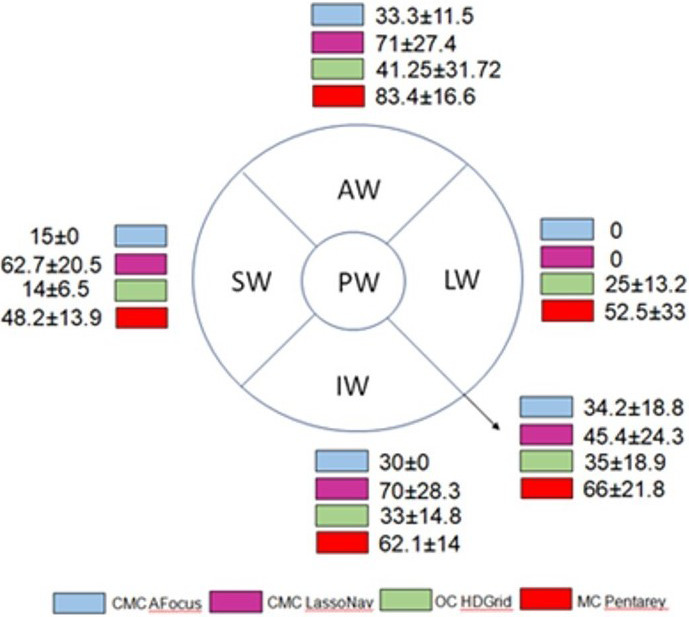
Extension and location of LVZs: comparison of the different catheters used. Extension and location of low‐voltage zones (LVZs) identified with circular catheters (CMCs) AFocus and Lasso Nav, omnipolar catheter (OC), and multipolar catheter (MC) in the different anatomical areas analyzed. LVZs have been quantified and calculated in square centimeters through the measurements yielded by the 3D mapping system used. The values are expressed as mean ± SD. AW, anterior wall; IW, inferior wall; LW, lateral wall; PW, posterior wall; SW, septal wall

**FIGURE 7:**
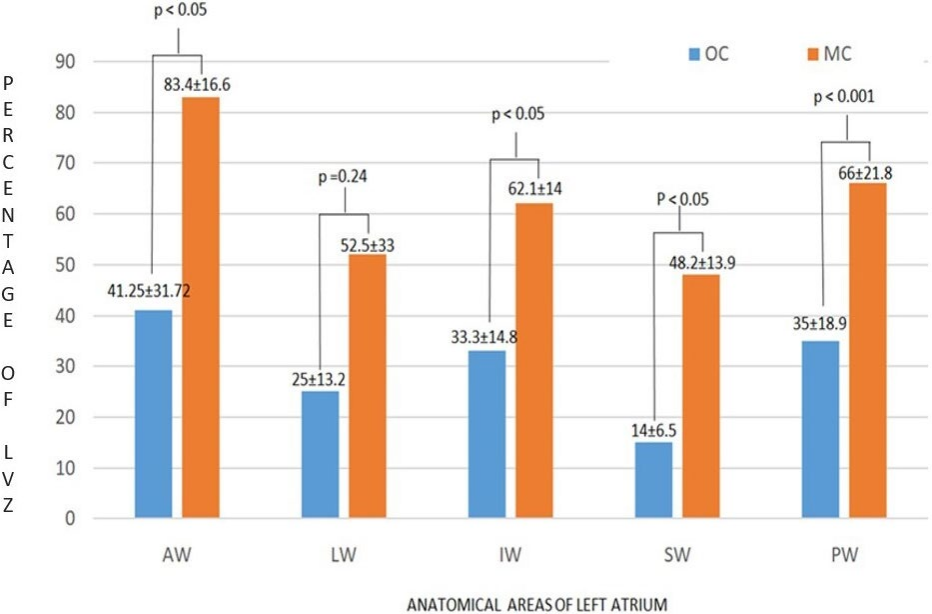
LVZs: comparison of OC versus MC. LVZs have been quantified and expressed as a percentage of the surface of each left atrial anatomical area analyzed. OC identified a significantly lower percentage of LVZs than MC in all the anatomical areas analyzed, with the exception of the LW. AW, anterior wall; IW, inferior wall; LW, lateral wall; MC, multipolar catheter; OC, omnipolar catheter; PW, posterior wall; SW, septal wall

As no LVZs were found in the left atrial roof of any patient, this anatomical area was excluded from the analysis. Figure 6 reports the mean values of LVZs expressed in square centimeters in each left atrial anatomical area analyzed. No significant differences were found between the LVZs identified by the two CMCs, while a lower percentage of LVZs was identified by OC than by MC in all the anatomical areas analyzed, except for the lateral wall (Figure 7).

## 4 DISCUSSION

In several recent studies, left atrial bipolar endocardial voltage maps constructed by acquiring thousands of voltage points by means of 3d‐S^14^ have emerged as a tool for defining AF substrates during RF ablation procedures.^15^ Indeed, LVZs has been considered a surrogate marker of the presence of atrial fibrosis, and may play a role in giving rise to the mechanisms underlying AF, especially in the case of persistent AF.^16^, ^17^ In these studies, however, mapping strategies and definitions of LVZs were heterogeneous. The electrical signals recorded by catheters are converted by 3d‐S into color‐coded voltage maps. These maps, however, may vary according to the catheters and 3d systems used. The problem is that several nonsubstrate factors can theoretically influence electrogram voltage.^18^, ^19^ Activation direction, electrode spacing, electrode size, filter settings, point density, and tissue contact are all factors that potentially influence HD maps; the technical challenges raised by these factors were discussed in a very interesting paper by Sim et al.^20^ In particular, the relationship between the orientation of the recording bipole and the wave‐front bipole may influence the arrival time of the activating wave‐front at each electrode. A solution to this problem may be provided by a new mapping technique, known as OC mapping; this is based on the use of a 3d mapping system and a multi‐electrode catheter that allows simultaneous recordings of unipolar electrograms, and which spans 2d and 3d space to derive conduction velocity and wave‐front direction.^21^ In this way, OC mapping enables detailed characterization of myocardial activation that is insensitive to catheter orientation; thus, the use of OC and 3d‐S EnSite Precision may be able to improve the detection of abnormal atrial substrate by using electrogram amplitude characteristics. Identifying LVZs also depends on the recording window specified for analysis and on the related voltage thresholds chosen for the definition of low voltages, since these factors can cause HD maps to vary markedly. Data on the relationships between pre‐ablation atrial fibrosis and atrial voltage thresholds are not currently available, and no true voltage threshold for atrial anomalies has been established. However, most studies have used a voltage amplitude <0.05 mV to identify scar areas and a value of >0.5 mV to identify normal tissues, while the range between 0.05 and 0.5 mV is commonly used to define the low voltages that identify the presence of underlying anomalies in the atrial structure. In our study, we chose the 0.05‐0.5 mV range adopted in several studies that have used 3d‐S CARTO and 3d‐S EnSite Precision, in order to construct HD maps through the recording of electrical signals acquired from the CMC Lasso Nav, CMC AFocus, or MC. For HD maps acquired by means of 3d‐S EnSite Precision and OC, we identified a range of LVZs that was more suited to representing atrial fibrosis. This was because 3d‐S EnSite Precision and OC differ from 3d‐S CARTO and MC in terms of their ability to select the bipolar electrograms. Indeed, by comparing different orientations of bipolar electrograms from the HD Grid electrode, OC electrograms acquired by means of OC match those of the largest bipolar electrogram, thereby eliminating the influence of reduced amplitude because of activation direction.^11^, ^13^ This relative increase in electrogram amplitude has been shown to change the voltage threshold by which tissue can be histologically defined as scar. This concept was demonstrated by a recent study on ventricular myocardial voltage, which reported that, when performing OC mapping, adopting a scar threshold of 1.5 mV (a value far higher than the standard 0.05 mV for conventional bipolar mapping) corresponded better to electrophysiologist‐determined scar than the area determined from bipolar signals.^22^ Additionally, Takigawa et al explored the optimal threshold in two configurations of OC in six infarcted sheep, and compared the impact of electrode spacing and bipolar direction on the scar threshold.^23^ They found that, although scar areas were well distinguished from areas of healthy tissues in any bipolar configurations with OC, bipolar spacing of 1 mm showed relatively lower accuracy and bipolar voltages generally increased as the inter‐electrode spacing increased in both healthy and scar areas. It has also been shown that LVZs documented by HD Grid and orthogonal HD‐wave configurations during VT ablation procedures are smaller than the same areas identified by means of the along and across bipolar configuration.^24^ Substrates in the left atrium of patients with AF are probably similar to, but not the same as, scar tissue because of infarction of the ventricular myocardium. It has been proposed that reentry mechanisms do not occur in densely fibrotic areas or scar tissue because there are not enough cardiomyocytes to allow impulse propagation,^25^ nor does it occur in mostly normal tissue, because any reentry circuits that are established are unstable and self‐terminate promptly. We identified anatomical areas characterized by SDC (red/yellow/green/light‐blue/blue). To do so, we used the best duplicate algorithm of 3d‐S EnSite Precision after setting an optimal voltage range and the OC HD Grid of 16 electrodes with 3 mm inter‐electrode spacing, which is the optimal spacing according to the results of the study by Takigawa. In our view, this approach offers a simple method of searching for potential and selective electro‐anatomical substrates underling AF, and probably constitutes the basis of a truly tailored ablation. Indeed, our approach, which was both qualitative and quantitative, helped us to identify potential patchy fibrosis; thus, it was probably able to define a substrate that could represent local non‐PV conductive alterations that influence anisotropy. This substrate probably plays a critical role in giving rise to AF mechanisms, as described by some authors.^26^, ^27^ We used the OC to search for the range that could best identify this substrate. This revealed that increasing the range from 0.05‐0.5 mV to 0.5‐1 mV increased the possibility of observing electrical alterations compatible with patchy fibrosis. By contrast, if bipolar signals are used, this capacity is lost, as scar tissue is more evident, and this probably does not express true electrical alterations. Modifying further the voltage window within the range 0.5‐1.0 mV, we observed a reduction in the amplitude of the substrate potential and limiting the LVZs with the range 0.3‐0.6 mV probably we identified the non‐PV diseased left atrial tissue more selectively. In fact, the voltage values in the ranges 0.3‐0.8 and 0.3‐0.6 have obviously remained the same and compatible with LVZs, but the minimum extension represented in the map with range 0.3‐0.6 tends to misleading representations of LVZs in different ranges. Finally, the results from our quantitative analysis of the substrate identified by means of OC and the optimal range were similar to those reported by Masuda et al.^28^ These authors compared along and across CMC bipolar HD maps with OC HD maps, and found a significantly lower percentage of LVZs in the majority of left atrial anatomical areas studied by means of OC than in those evaluated by means of bipolar HD maps. In our experience, the total number of voltage points acquired were significantly higher with OC than with MC, thus allowing us to construct maps with higher density. These findings are also in line with those of Masuda. Moreover, the procedural time needed for the acquisition of voltage points was similar among the different catheters used; this was probably because of the fact that more complex mapping is required in patients with persistent AF, in whom OC were preferentially used, while CMC mapping yielded fewer voltage point.

### 4.1 Limitations

This study has some limitations. Firstly, it was single‐center and retrospective; future studies should be prospective and multicenter and include direct comparison of diagnostic catheter technologies in a randomized setting. However, although a direct comparison between the various catheter technologies in the same patient would have been preferable, it was not performed because it was unethical. Moreover, only a small number of patients were included, while larger series are needed in order to fully assess the efficacy of 0.3 mV as the lower and 0.6 mV as the upper cut‐off limits when OC are used to identify substrates that may play a role in maintaining AF. In addition, the choice of the 0.3‐0.6 range to identify LVZs more accurately was based on our interpretation that larger LVZs may be more representative of false identifications. A comparison between MRI assessments, that we did not perform in this case, and patchy fibrosis identified with maps in the 0.3‐0.6 mV range could provide further information on the worth of our range. Finally, our search for LVZs was arbitrarily performed only during sinus rhythm and no comparison was made during AF; the superiority of the sinus rhythm approach therefore remains to be validated. Finally, we did not evaluate the impact, in terms of outcome, of tailored RF ablation of the substrates identified by means of OC in comparison with a matched control group of patients in whom the substrates were identified by means of MC and this may be grounds for further studies in the future.

## 5 CONCLUSION

According to our experience, the combined use of OC and 3d‐S EnSite Precision selectively identifies substrates that are potentially responsible for the mechanisms of AF by detecting LVZs that are not affected by the orientation of the catheter with respect to the activating wave‐front. This approach therefore overcomes one of the limitations of using HD maps derived from bipolar catheters. In our study, the 0.3‐0.6 mV range identified potential fibrotic substrates that could play a role in the mechanism of sustained AF better than the standard range of 0.05‐0.5 mV. However, further studies are needed in order to determine whether 0.3‐0.6 mV is the optimal range within which to identify LVZs as expression of patchy fibrosis by means of HD Grid and EnSite Precision.

## References

[1] Haïssaguerre M , Jaïs P , Shah DC , et al. Spontaneous initiation of atrial fibrillation by ectopic beats originating in the pulmonary veins. N Engl J Med. 1998;339(10):659–66. https://www.nejm.org/doi/full/10.1056/NEJM199809033391003 9725923 10.1056/NEJM199809033391003

[2] Shiroshita‐Takeshita A , Brundel BJ , Nattel S . Atrial fibrillation: basic mechanisms, remodeling and triggers. J Interv Card Electrophysiol. 2005;13(3):181–93. https://link.springer.com/article/10.1007/s10840‐005‐2362‐y 16177845 10.1007/s10840-005-2362-y

[3] Calkins H , Hindricks G , Cappato R , et al. HRS/EHRA/ECAS/APHRS/SOLAECE expert consensus statement on catheter and surgical ablation of atrial fibrillation. Heart Rhythm. 2017;14(10):e275–e444. 10.1016/j.hrthm.2017.05.012 28506916 PMC6019327

[4] H. Lau D , Linz D , Schotten U , Mahajan R , Sanders P , Kalman JM Pathophysiology of paroxysmal and persistent atrial fibrillation: rotors, foci and fibrosis Heart Lung Circul. 2017. https://www.ncbi.nlm.nih.gov/pubmed/28610723 10.1016/j.hlc.2017.05.119 28610723

[5] Marrouche NF , Wilber D , Hindricks G , et al. Association of atrial tissue fibrosis identified by delayed enhancement MRI and atrial fibrillation catheter ablation: the DECAAF study. JAMA. 2014;311(5):498. https://www.ncbi.nlm.nih.gov/pubmed/24496537 24496537 10.1001/jama.2014.3

[6] Tschabrunn CM , Roujol S , Dorman NC , Nezafat R , Josephson ME , Anter E . High‐resolution mapping of ventricular scar: comparison between single and multielectrode catheters. Circ Arrhythm Electrophysiol. 2016;9(6). https://www.ahajournals.org/doi/abs/10.1161/circep.115.003841 10.1161/CIRCEP.115.003841 PMC4911826 27307518

[7] Rolf S , Kircher S , Arya A , et al. Tailored atrial substrate modification based on low‐voltage areas in catheter ablation of atrial fibrillation. Circ Arrhythm Electrophysiol. 2014;7(5):825–833. https://www.ahajournals.org/doi/full/10.1161/circep.113.001251 25151631 10.1161/CIRCEP.113.001251

[8] Jadidi AS , Lehrmann H , Keyl C , et al. Ablation of persistent atrial fibrillation targeting low‐voltage areas with selective activation characteristics. Circ Arrhythm Electrophysiol. 2016;9(3). https://www.ahajournals.org/doi/full/10.1161/CIRCEP.115.002962 10.1161/CIRCEP.115.002962 26966286

[9] Kottkamp H . Human atrial fibrillation substrate: towards a specific fibrotic atrial cardiomyopathy. Eur Heart J. 2013;34(35):2731–8. 10.1093/eurheartj/eht194 23761394

[10] Kircher S , Arya A , Altmann D , et al. Individually tailored vs. standardized substrate modification during radiofrequency catheter ablation for atrial fibrillation: a randomized study. EP Europace. 2018;20(11):1766–75. 10.1093/europace/eux310 29177475

[11] Haldar SK , Magtibay K , Porta‐Sanchez A , et al. Resolving bipolar electrogram voltages during atrial fibrillation using omnipolar mapping. Circ Arrhythm Electrophysiol. 2017. https://www.ncbi.nlm.nih.gov/pubmed/28887362 10.1161/CIRCEP.117.005018 28887362

[12] Yamagushi T , Fukui A , Node K . Bipolar voltage mapping for the evaluation of atrial substrate: can we overcome the challenge of directionality? J Atr Fibrillation. 2019. https://www.ncbi.nlm.nih.gov/pmc/articles/PMC6533827 10.4022/jafib.2116 PMC6533827 31139298

[13] Massé S , Magtibay K , Jackson N , et al. Resolving myocardial activation with novel omnipolar electrograms. Circ Arrhythm Electrophysiol. 2016;9:e004107. 10.1161/CIRCEP.116.004107 27406608 PMC4956680

[14] Kapa S , Desjardins B , Callans DJ , Marchlinski FE , Dixit S . Contact electroanatomic mapping derived voltage criteria for characterizing left atrial scar in patients undergoing ablation for atrial fibrillation. J Cardiovasc Electrophysiol. 2014;25:1044–52. 10.1111/jce.12452 24832482

[15] Andronache M , Drca N , Viola G . High‐resolution mapping in patients with persistent AF. AER Journal https://. 2019;8(2):111–5. 10.15420/aer.2018.57.1 PMC6528045 31114685

[16] Tzeis S , Asvestas D , Vardas P . Atrial fibrosis: translational considerations for the management of AF patients. AER J. 2019;8(1):37–41. https://www.aerjournal.com/articles/atrial‐fibrosis‐translational 10.15420/aer.2018.79.3 PMC6434500 30918665

[17] Hunter RJ , Liu Y , Lu Y , Wang W , Schilling JR . Left atrial wall stress distribution and its relationship to electrophysiologic remodeling in persistent atrial fibrillation. Circ Arrhythm Electrophysiol. 2012. https://www.ahajournals.org/doi/abs/10.1161/circep.111.965541 10.1161/CIRCEP.111.965541 22294615

[18] Josephson ME , Anter E . Substrate mapping for ventricular tachycardia. Assumptions and misconceptions. JACC Clin Electrophysiol. 2015. https://www.ncbi.nlm.nih.gov/pubmed/29759461 10.1016/j.jacep.2015.09.001 29759461

[19] Anter E , Tschabrunn CM , Josephson ME . High‐resolution mapping of scar‐related atrial arrhythmias using smaller electrodes with closer interelectrode spacing. Circ Arrhythm Electrophysiol. 2015;8(3):537–45. https://www.ncbi.nlm.nih.gov/pubmed/25792508 25792508 10.1161/CIRCEP.114.002737

[20] Sim I , Bishop M , O’Neill M , Williams SE . Left atrial voltage mapping: defining and targeting the atrial fibrillation substrate. J Int Card Electrophysiol. 2019;56(3):213–27. https://link.springer.com/article/10.1007/s10840‐019‐00537‐8 10.1007/s10840-019-00537-8 PMC6900285 31076965

[21] Deno DC , Balachandran R , Morgan D , Ahmad F , Masse S , Nanthakumar K . Orientation‐independent catheter‐based characterization of myocardial activation. IEEE Trans Biomed Eng. 2017;64(5):1067–77. https://www.ncbi.nlm.nih.gov/pubmed/27411215 27411215 10.1109/TBME.2016.2589158

[22] Magtibay K , Massé S , Asta J , et al. Physiological assessment of ventricular myocardial voltage using omnipolar electrograms. J Am Heart Assoc. 2017;6(8). https://www.ncbi.nlm.nih.gov/pubmed/28862942 10.1161/JAHA.117.006447 PMC5586472 28862942

[23] Takigawa M , Relan J , Kitamura T , et al. Impact of spacing and orientation on the scar threshold with a high‐density grid catheter. Circ Arrhythm Electrophysiol. 2019;12(9). https://www.ahajournals.org/doi/10.1161/CIRCEP.119.007158 10.1161/CIRCEP.119.007158 31446771

[24] Okubo K , Frontera A , Bisceglia C , et al. Grid mapping catheter for ventricular tachycardia ablation. Circ Arrhythm Electrophysiol. 2019;12(9). https://www.ahajournals.org/doi/10.1161/CIRCEP.119.007500 10.1161/CIRCEP.119.007500 31500436

[25] Vigmond E , Pashaei A , Amraoui S , Cochet H , Haissaguerre M . Percolation as a mechanism to explain atrial fractionated electrograms and reentry in a fibrosis model based on imaging data. Heart Rhythm. 2016;13(7):1536–43. https://www.ncbi.nlm.nih.gov/pubmed/26976038 26976038 10.1016/j.hrthm.2016.03.019

[26] Nattel S . Molecular and cellular mechanisms of atrial fibrosis in atrial fibrillation. JACC Clin Electrophysiol. 2017;3(5):425–35. https://electrophysiology.onlinejacc.org/content/3/5/425. 29759598 10.1016/j.jacep.2017.03.002

[27] Zahid S , Boyle PM , Schwarz EL , et al. Patient‐derived models link re‐entrant driver location in atrial fibrillation to fibrosis spatial pattern. Cardiovasc Res. 2016. https://academic.oup.com/cardiovascres/article/110/3/443/1744836 10.1093/cvr/cvw073 PMC4872878 27056895

[28] Masuda M , Asai M , Iida O , et al. Left atrial voltage mapping with a direction‐independent grid catheter: comparison with a conventional circular mapping catheter. J Cardiovasc Electrophysiol. 2019. https://www.ncbi.nlm.nih.gov/pubmed/31701587 10.1111/jce.14263 31701587

